# RACIAL–ETHNIC DISPARITIES IN STRESS EXPOSURE AND THE ONSET OF LATER-LIFE LIMITATIONS IN ACTIVITIES OF DAILY LIVING

**DOI:** 10.1093/geroni/igad104.1013

**Published:** 2023-12-21

**Authors:** Madison Sauerteig-Rolston, Kenneth Ferraro

**Affiliations:** Purdue University, West Lafayette, Indiana, United States; Purdue University, West Lafayette, Indiana, United States

## Abstract

This study investigates the relationship between stress exposure – including cumulative stress burden and domain-specific stressors – and the onset of activities of daily living (ADL) limitations in later life and examines whether there are racial-ethnic and nativity differences in this relationship. Examining data from the Health and Retirement study (HRS), we apply logistic regression and Weibull accelerated failure-time models to examine associations between stress and baseline ADL limitations as well as stress exposure and age of onset of ADL limitations over a decade. Interaction tests between stress and race-ethnicity and nativity were employed to understand how stress exposure uniquely influences ADL limitations among White, Black, US-born Hispanic, and foreign-born Hispanic older adults. Black, US-born Hispanic, and foreign-born Hispanic adults were more likely to develop an ADL limitation at a younger age compared to White adults (β = -0.06; β = -0.07; β = -0.09; respectively). Cumulative stress burden, childhood traumatic events, adult financial strain, everyday discrimination, and major lifetime discrimination were associated with an earlier onset of ADL limitation among White, Black, US-born Hispanic and foreign-born Hispanic adults. Further, US-born Hispanic adults that experienced the same amount of childhood traumatic events as White adults experienced an earlier transition to ADL limitation by a factor of 0.65 or in about 35% of the time. Reducing racial-ethnic and nativity disparities in ADL limitations in later-life will require efforts to adjudicate the long lasting historical and structural disadvantages that contribute to differential stress exposure across the life course.

